# The Natural Flavonoid Compound Deguelin Inhibits HCMV Lytic Replication within Fibroblasts

**DOI:** 10.3390/v10110614

**Published:** 2018-11-07

**Authors:** Masatoshi Nukui, Christine M. O’Connor, Eain A. Murphy

**Affiliations:** 1 Genomic Medicine, Lerner Research Institute, Cleveland Clinic, Cleveland, OH 44106, USA; nukuim@ccf.org; 2 FORGE Life Science, Pennsylvania Biotechnology Center, Doylestown, PA 18901, USA

**Keywords:** cytomegalovirus, HCMV, deguelin, ganciclovir

## Abstract

Human cytomegalovirus (HCMV) is a ubiquitous herpesvirus for which there is no vaccine or cure. This viral infection, once acquired, is life-long, residing latently in hematopoietic cells. However, latently infected individuals with weakened immune systems often undergo HCMV reactivation, which can cause serious complications in immunosuppressed and immunocompromised patients. Current anti-viral therapies target late stages of viral replication, and are often met with therapeutic resistance, necessitating the development of novel therapeutics. In this current study, we identified a naturally-occurring flavonoid compound, deguelin, which inhibits HCMV lytic replication. Our findings reveal that nanomolar concentrations of deguelin significantly suppress the production of the infectious virus. Further, we show that deguelin inhibits the lytic cycle during the phase of the replication cycle consistent with early (E) gene and protein expression. Importantly, our data reveal that deguelin inhibits replication of a ganciclovir-resistant strain of HCMV. Together, our findings identify a novel, naturally occurring compound that may prove useful in the treatment of HCMV replication.

## 1. Introduction

Human cytomegalovirus (HCMV) is a betaherpesvirus that infects a majority of the human population. Like all herpesviruses, once an individual is infected, HCMV remains with its host for life, residing in a latent state in cells of the hematopoietic compartment. With the exception of specific subpopulations including fetuses, neonates, stem cell recipients and sero-negative organ transplant recipients, primary HCMV infection poses little threat to otherwise healthy individuals. However, when an infected individual’s immune system is severely compromised, HCMV can reactivate from latency, which results in lytic replication, viral spread, and disease that can be fatal.

While there is no vaccine or cure for HCMV, current anti-viral treatments do exist [1]. A majority of the commonly prescribed HCMV antiviral therapies are nucleoside apologues that require active HCMV *UL97*, a gene that encodes a serine/threonine kinase, pUL97. This HCMV-encoded kinase phosphorylates nucleoside analogs such, as ganciclovir, thereby converting it to a competitive inhibitor of dGTP incorporation into viral DNA by the HCMV encoded DNA polymerase UL54, thus resulting in robust anti-viral activity [2]. Similarly, valganciclovir is also phosphorylated by pUL97, but has increased absorption and bioavailability compared to ganciclovir, due to an l-valyl ester added to the 5′-end of ganciclovir’s deoxyribose ring [3]. More recently, maribavir emerged as a promising, new anti-viral, which competes with ATP binding to pUL97, thereby inhibiting its kinase activity [4]. However, these therapies, while potent inhibitors of HCMV, are fraught with problems. Clinicians treating patients suffering from HCMV reactivation are noting a significant increase in drug-resistant HCMV strains, due specifically to *UL97* mutations. While maribavir seemed promising as an alternative treatment strategy for patients with ganciclovir- or valganciclovir-resistant HCMV, it is now evident that this therapy also leads to drug resistant HCMV.

Drug resistant viral reactivation requires turning to alternative treatments, including foscarnet and cidofovir. Foscarnet, a pyrophosphate analog, is a DNA polymerase inhibitor, but is not activated by pUL97 [5]. Similarly, cidofovir is also a DNA polymerase inhibitor, but unlike the nucleoside analogs ganciclovir or valganciclovir, cidofovir activity does not require phosphorylation by pUL97 [6]. Though these additional drugs seemed promising for treating patients with drug-resistant HCMV, it is now clear that these treatments are also leading to multi-drug resistant strains. Additionally, even in cases where the patient does not become resistant, foscarnet is highly nephrotoxic [7] and cidofovir is associated with severe neutropenia [8]. Recently a viral terminase inhibitor, letermovir, was approved to prophylactically treat hematopoietic cell transplant patients, and recent clinical data suggest that this prophylaxis results in a significant decrease in clinically significant HCMV infection in these patients [9]. However, in vitro testing of the terminase complex suggests that letermovir-resistant strains of HCMV may emerge [10,11]. In sum, detrimental side effects of current anti-HCMV treatments, coupled with increasingly hard to manage drug resistant HCMV strains, underscore the need for novel therapeutics that combat HCMV.

In this current study, we show that the naturally occurring compound, deguelin, significantly inhibits HCMV lytic replication. Deguelin [(7aS, BaS)-13, 13a-dihydro-9, 10-dimethoxy-3, 3-dimethyl-3H-*bis* [1] benzo-prano [3, 4-b: 6′, 5′-e] pyran-7 (7aH)-one] is a plant-derived rotenoid compound and is a member of the flavonoid family. This compound has shown anti-cancer activities in pre-clinical trials [12], as well as promoting cell cycle arrest, apoptosis, and inhibiting angiogenesis. Additionally, deguelin blocks phosphoinositides 3-kinase (PI3K)/Akt activity and binds to the ATP binding domain of Heat Shock Protein 90α (HSP90), all pathways critical for efficient viral infection. Herein, our results reveal that low concentrations of deguelin are effective at inhibiting HCMV by targeting viral early (E) protein expression, thereby reducing the production of infectious virus. These findings suggest that deguelin could prove a novel therapeutic for HCMV lytic infection.

## 2. Materials and Methods

### 2.1. Cells, Viruses, and Compounds

Primary newborn human fibroblasts, NuFF-1 cells (GlobalStem, Rockville, MD, USA), were maintained in Dulbecco’s modified Eagle medium (DMEM), supplemented with 10% fetal bovine serum (FBS), 2 mM l-glutamine, 0.1 mM non-essential amino acids, 10 mM 4-(2-hydroxyethyl)-1-piperazineethanesulfonic acid (HEPES), and 100 U/mL each of penicillin and streptomycin. Cells were grown at 37 °C in 5% CO_2_.

Bacterial artificial chromosome (BAC)-derived TB40/E*mCherry* [13] and TR*gfp* [14] are described elsewhere. The recombinant virus TB40/E*mCherry*-UL99eGFP (UL99eGFP) [15] was generated by tagging the C-terminus of the UL99 open reading frame (ORF) with enhanced green fluorescent protein (eGFP) with the forward (FOR) and reverse (REV) primers listed in Table 1 using standard galK recombineering protocols, described in detail previously (e.g., Refs. [13,16,17]). The resultant viral recombinant was sequenced to verify the seamless in-frame insertion of eGFP upstream of the UL99 stop codon. To generate virus stocks, each of the aforementioned viruses were grown as previously described (e.g., Ref. [13]). All viral stocks were titered by 50% tissue culture infectious dose (TCID_50_) assay on NuFF-1 cells.

The Spectrum collection of compounds (MicroSource Discovery Systemts, Inc.) was used to treat NuFF-1 cells (50 μM) for 2 hours (h) prior to infection with TB40/E*mCherry*-UL99eGFP at a multiplicity of infection (moi) = 0.01 for 72 h. Viral replication was measured using mCherry (a marker of infection) and eGFP (a marker of late viral replication) fluorecence, analyzed with an Operetta High Content Imaging System (PerkinElmer, Waltham, MA, USA). Deguelin and ganciclovir were both purchased from Cayman. Deguelin and ganciclovir were reconstituted in dimethyl sulfoxide (DMSO). Deguelin was used at various concentrations, as indicated throughout the text, and ganciclovir was used at 10 μM.

### 2.2. Cytotoxicity and Cytostaticity Assays

Cytotoxic effects of deguelin were measured by treating confluent NuFF-1 cells with varying concentrations of deguelin for 3 days (d), as indicated. Cell proliferation was measured using the CellTiter 96 AQueous One Solution Cell Proliferation Assay (Promega, Madison, WI, USA), in accordance to the manufacturer’s instructions. Absorbance was read at 490 nm using a Cytation 3 cell imaging plate reader (BioTek, Winooski, VT, USA). Data is plotted as percent inhibition relative to vehicle (DMSO)-treated cells. Cell doubling was assessed by seeding NuFF-1 cells at 25% confluence and treating with varying concentrations of deguelin, as described in the text. After two cell doublings, cell proliferation was measured using CellTiter 96 AQueous One Solution Cell Proliferation Assay (Promega), as described above. Drug-treated cells were normalized to vehicle (DMSO)-treated cells.

### 2.3. Half-Maximal Inhibitory Concentration (IC_50_) Measurements

NuFF-1 cells were infected in the presence of varying concentrations of degeulin at either a high multiplicity of infection (moi) of 1.0 or low moi of 0.01 for 7 or 14 d, respectively. Cell-associated DNA was isolated by phenochloroform-isoamyl alcohol precipitation, as previously described [18], for quantitative PCR (qPCR) analyses (described in Section 2.4) and viral supernatants were collected for TCID_50_ analyses. IC_50_ was determined using the on-line calculation tool: https://www.aatbio.com/tools/ic50-calculator/.

### 2.4. Viral RNA, DNA, and Protein Analyses

NuFF-1 cells were treated with deguelin (250 nM) or vehicle (DMSO) at the indicated concentrations followed by infection with HCMV at multiplicities and durations indicated in the text for subsequent RNA, DNA, or protein analyses. Total RNA was harvested, and viral transcripts were measured by reverse transcriptase quantitative PCR (RTqPCR) using the primers listed in Table 1. Viral transcripts were normalized to cellular GAPDH RNA levels. To assess viral DNA, cell-associated DNA was harvested and assessed by qPCR using primers directed at UL122 (Table 1). All samples were normalized to cellular MDM2 (Table 1). To determine viral protein production, cell lysates were collected as indicated in the text and proteins were separated by SDS-PAGE and then transferred to nitrocellulose using a semi-dry transfer apparatus (Owl). The following antibodies were used: anti-IE2 (clone #3A9; Ref. [19]) and anti-pp28 (clone #10B4-29; Ref. [20]), each diluted at 1:1000; anti-pUL44 (Virusys; diluted 1:10,000); and anti-alpha-tubulin (Sigma, St. Louis, MO, USA; diluted 1:5000). In all cases, membranes were incubated with IR Dye 680RD anti-mouse secondary antibody (1:15,000; LI-COR, Lincoln, NE, USA) and scanned using an Odyssey Imaging System (LI-COR). Protein bands were visualized by Image Studio Software (LI-COR). Fluorescence images were captured using an Olympus (Shinjuku, Tokyo, Japan) IX8 microscope and images were processed using SlideBook 5.0 software (3i, Denver, CO, USA).

## 3. Results

### 3.1. Deguelin Treatment Inhibits HCMV Lytic Replication

**Figure 1:**
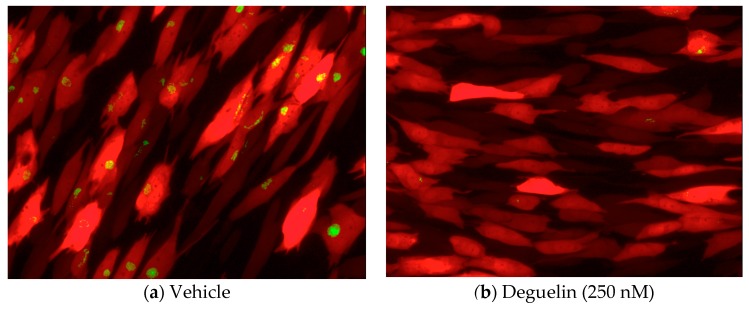
Deguelin significantly reduces the expression of late gene products in HCMV-infected NuFF-1 cells. NuFF-1 cells were infected with TB40/E*mCherry*-UL99eGFP (UL99eGFP; moi = 1.0) in the presence of (**a**) vehicle (DMSO) or (**b**) deguelin (250 nM) for 72 h. Red, mCherry (marker of infection); green, eGFP (pp28 expression).

To identify novel FDA- or internationally-approved drugs that possess off-label antiviral activity against HCMV, we analyzed viral replication in fibroblasts treated with compounds from the commercially available Spectrum library (MicroSource Discovery Systems, Inc., Gaylordsville, CT, USA). We pretreated NuFF-1 cells with 50 μM of each compound for 2 hours (h), after which we infected these cells with TB40/E*mCherry*-UL99eGFP (UL99eGFP) for 72 h at a multiplicity of 0.01 TCID_50_/mL. Within this virus, eGFP is fused in frame to the UL99 ORF, which encodes pp28, a protein that functions during HCMV assembly. Expression of the UL99eGFP fusion is controlled by the virus’ endogenous UL99 promoter, thus providing an easily identifiable late kinetic fluorescent marker to monitor viral replication in the compound library screen assay. We also included ganciclovir treatment, since it is a well-established HCMV anti-viral and thus served as an internal control for the screen. We prioritized compounds whose treatment resulted in a significant reduction in pp28, as measured by eGFP (Supplemental Table 1). One of the compounds that caused effective inhibition of pp28 fusion protein expression was deguelin (Spectrum). Thus, we focused our efforts to understanding the impact of deguelin on HCMV replication. First, we confirmed the above finding by pre-treating NuFF-1 cells with either vehicle (DMSO) or deguelin (250 nM) for 1 h prior to infection with UL99eGFP (moi = 1.0). At 72 hpi, we visualized each infected cell population by immunoflourescence and confirmed that deguelin treatment significantly reduced the expression of the viral late protein encoded by UL99, pp28, fused in-frame to eGFP, whose expression occurs only after efficient viral DNA replication (Figure 1) similar to the published subcellular localization of untagged UL99 [20].

**Figure 2:**
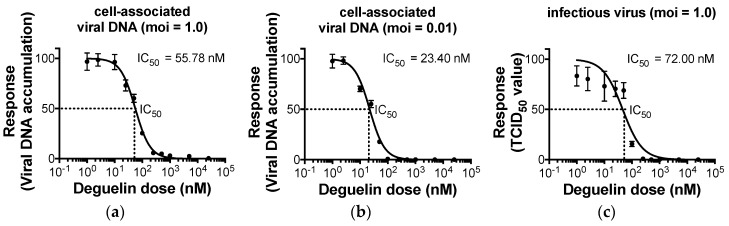
Deguelin suppresses HCMV replication in infected fibroblasts. NuFF-1 cells were infected with UL99*eGFP* in the presence of vehicle (DMSO) or deguelin at either (**a**) a high multiplicity (moi = 1.0) for 7 d, or (**b**) a low multiplicity (moi = 0.01) for 14 d. Cell-associated DNA was harvested from the respective cultures and analyzed by qPCR using primers directed at UL122. Samples were analyzed in triplicate and normalized to cellular MDM2. (**c**) NuFF-1 cells were infected as in (**a**), and at 7 dpi, the cell-free virus was harvested and viral titers were analyzed by TCID_50_. For all panels, error bars represent the standard deviation of three technical replicates. Replicate assays were performed (*n* = 3), and a representative biological replicate is shown in each panel.

Herpesvirus lytic replication is a highly coordinated phase of infection, during which viral genes are expressed in a synchronized cascade. Immediate early (IE) genes are transcribed first, whose proteins are involved in immune evasion and facilitate the expression of early (E) genes. E proteins facilitate viral DNA replication, which once completed, triggers the expression of L genes and their proteins, which encode virion components and facilitate viral egress. If any of these stages are impeded, the following step in the cascade is also impacted. Since we observed deguelin treatment inhibited pp28 expression, we next asked if viral DNA replication is impacted by deguelin and at what concentration. To this end, we pretreated NuFF-1 cells for 1 h with deguelin at varying concentrations and then infected these cells with UL99*eGFP* at a high (moi = 1.0; 7 d) or low (moi = 0.01; 14 d) multiplicity. At each respective time point, we assessed the cell-associated viral DNA by qPCR for the viral genome, and then calculated the deguelin concentration at which viral DNA is reduced by half (IC_50_). Our results show that deguelin treatment significantly reduces HCMV cell-associated viral DNA at both multiplicities tested. We found that for the high moi, the IC_50_ concentration for deguelin was 55.8 nM, whereas the deguelin IC_50_ value for the low moi infection was 23.4 nM (Figure 2a,b). We anticipated a lower IC_50_ value for the low moi infection, as such growth curves required subsequent rounds of viral replication and infection.

**Figure 3:**
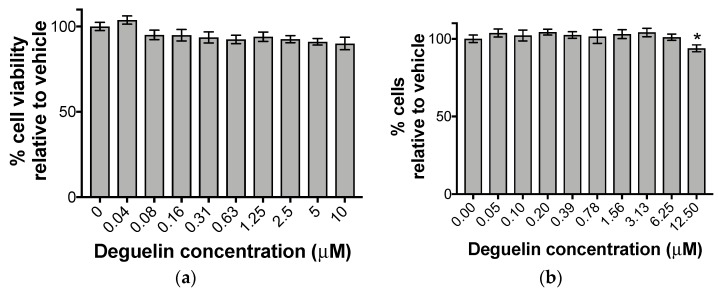
Deguelin treatment was not cytotoxic or cytostatic to fibroblasts. (**a**) NuFF-1 cells were grown to confluence and then treated with vehicle (DMSO) or increasing concentrations of deguelin, as indicated, for 72 h. Cell viability was then assessed by 3-(4,5-dimethylthiazol-2-yl)-2,5-diphenyltetrazolium bromide (MTT) assay. Data was recorded at 570 nm using a Cytation 3 plate reader. Data is presented as percent (%) inhibition relative to vehicle. (**b**) NuFF-1 cells were plated at 25% confluence and then treated with vehicle (DMSO) or increasing concentrations of deguelin. Cells were allowed to undergo two cell doublings, and were then assayed using the method used in (**a**). Data is presented as the number of cells relative to vehicle. Error bars represent the standard deviation of three technical replicates. Replicate assays were performed (*n* = 3), and a representative biological replicate is shown in each panel. Statistical analyses were performed using Student’s *t*-test between each indicated concentration relative to the vehicle-treated cells; * *p* < 0.01.

We reasoned that this significant reduction in cell-associated viral DNA would result in a significant impact on the production of the infectious virus. Hence, to determine the impact of deguelin on the release of extracellular, infectious virions, we pre-treated NuFF-1 cells with vehicle (DMSO) or varying concentrations of deguelin for 1 h, and then infected these cells with UL99*eGFP* (moi = 1.0) for 7 d. We then harvested the cell-free supernatant and determined the titer of virus released by TCID_50_ on naïve NuFF-1 cells. We found that as little as 72 nM was sufficient to inhibit infectious virion production by half (Figure 2c). If the compound adversely impacts the health of the host cell irrespective of viral infection, we would expect to observe a reduction in viral replication following drug treatment, thereby leading to a false-positive result when measuring the antiviral response. Thus, to determine the toxicity of deguelin on NuFF-1 cells in the absence of HCMV infection, we monitored cell viability by quantifying cellular metabolic activity as well as cell doubling times after treatment with deguelin in various concentrations. Importantly, we found that deguelin treatment was neither cytotoxic nor cytostatic to NuFF-1 cells at concentrations found to be inhibitory to HCMV replication (Figure 3a,b). Taken together, our in vitro findings show that deguelin inhibits HCMV lytic replication at concentrations that display little-to-no adverse effect on cell viability.

### 3.2. Deguelin Treatment Effectively Inhibits Ganciclovir-Resistant HCMV

**Figure 4:**
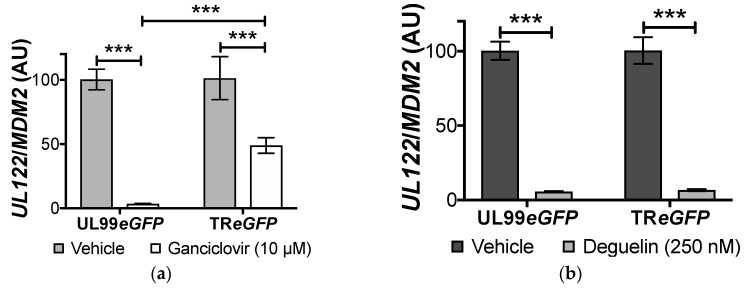
Ganciclovir-resistant HCMV is sensitive to deguelin treatment. NuFF-1 cells were treated with (**a**) ganciclovir (10 μM) or (**b**) deguelin (250 nM) for 1 h, after which cultures were infected with either TB40/E*mCherry*-UL99eGFP (UL99*eGFP*) or TR*eGFP* for 7 d (moi = 1.0). Vehicle-treated cells in each case received DMSO. Cell-associated viral DNA was assessed by qPCR using primers directed at *UL122*. Each sample was analyzed in triplicate and normalized to cellular *MDM2*. Data is presented as the ratio of *UL122*/*MDM2* relative to vehicle. Error bars represent the standard deviation of three technical replicates. Replicate assays were performed (*n* = 3), and a representative biological replicate is shown in each panel. AU = arbitrary units. Statistical significance was calculated by two-way ANOVA, *** *p* < 0.0001.

Nucleoside analogues are the standard of care treatment administered to patients undergoing HCMV reactivation, the most common of which is ganciclovir. However, some strains of HCMV are resistant to this anti-viral, rendering these patients refractive to this treatment. Ganciclovir resistance is attributed to mutations within the HCMV-encoded kinase, *UL97*, underscoring the need for novel therapies to suppress HCMV lytic replication. The reported effects of deguelin on cancer cells are mediated through host cell protein targeting and thus we predict that it does not require kinase phosphorylation to function. Therefore, we hypothesized that a ganciclovir-resistant HCMV strain would be sensitive to this drug. To determine the effectiveness of deguelin on inhibiting ganciclovir-resistant HCMV, we utilized TR*eGFP*. This BAC-derived strain lacks codons 591–594 of *UL97*, rendering it less responsive to ganciclovir treatment [21]. First, we pretreated NuFF-1 cells with the well-established effective dose of ganciclovir (10 μM) for 1 h. We then infected these cultures with either UL99*eGFP* (a TB40/E-derived recombinant) or TR*eGFP* for 7 d (moi = 1.0). We then measured viral DNA replication by quantifying viral genomes using qPCR. We confirmed that TR*eGFP* was less responsive to this treatment compared to UL99*eGFP* (Figure 4a). While, ganciclovir did inhibit TR*eGFP* by approximately 50 percent, UL99*eGFP* displayed almost 97% inhibition following ganciclovir treatment. However, when we repeated this experiment using deguelin, we found that viral DNA replication from both UL99*eGFP* and TR*eGFP* were significantly inhibited following a 250 nM deguelin treatment by approximately 95% (Figure 4b). These data suggest that deguelin inhibits ganciclovir-resistant HCMV.

### 3.3. Lytic Gene Transcription Is Inhibited by Deguelin Treatment

**Figure 5:**
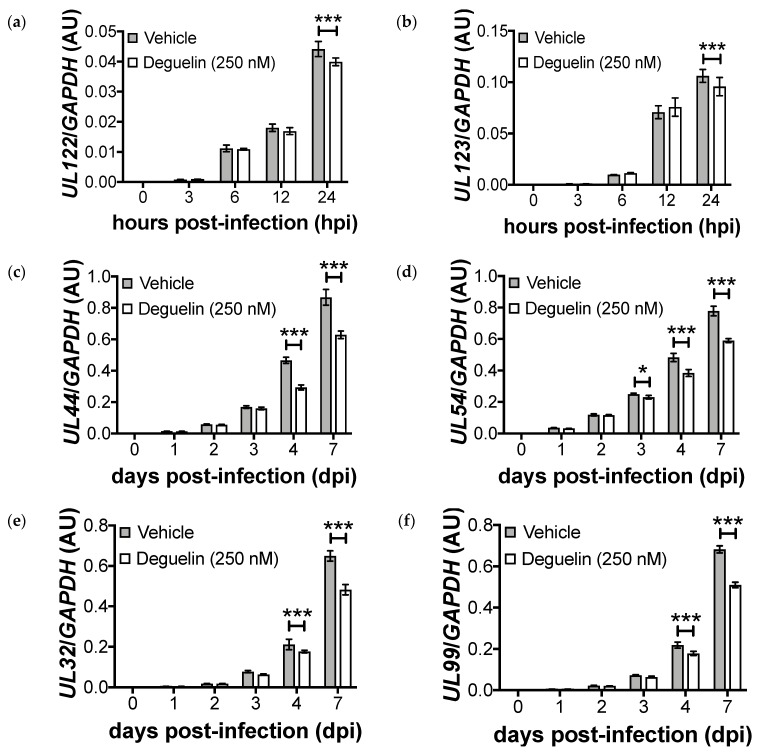
Treatment of HCMV infected fibroblasts with deguelin decreases IE, E, and L gene transcripts. NuFF-1 cells were treated with vehicle (DMSO) or deguelin (250 nM) for 1 h, after which cultures were infected with UL99*eGFP* (moi = 1.0). Total RNA was collected at the indicated time points. Viral transcripts were assessed by RTqPCR using the following primers: (**a**) *UL123* and (**b**) *UL122* (IE); (**c**) *UL44* and (**d**) *UL54* (E); (**e**) *UL32* and (**f**) *UL99* (L). Each sample was analyzed in triplicate and normalized to cellular *GAPDH*. Error bars represent the standard deviation of three technical replicates. Replicate assays were performed (*n* = 3), and a representative biological replicate is shown in each panel. AU = arbitrary units. Statistical significance was calculated by two-way ANOVA, * *p* < 0.05, ** *p* < 0.005, *** *p* < 0.0001.

Our data reveal that deguelin treatment impacts L viral protein expression (Figure 1) and viral DNA replication is significantly decreased in the presence of deguelin (Figure 2a,b). Our findings also indicate that deguelin treatment blocks the production of infectious virions (Figure 2c). To begin to understand the point during viral lytic replication at which deguelin functions, we pre-treated NuFF-1 cells with vehicle (DMSO) or deguelin (250 nM) and then infected these cells with UL99*eGFP* (moi = 1.0). We collected total RNA at the indicated time points and assessed two representative genes from each class of lytic gene transcription: *UL123* and *UL122* (IE), *UL44* and *UL54* (E), and *UL32* and *UL99* (L). We found that IE transcription was unaffected over the first 12 hpi in response to deguelin treatment (Figure 5a,b), though we noted a minor yet statistically significant difference by 24 hpi. However, we found that deguelin significantly impacted each E and L viral transcript we tested by 4 dpi (Figure 5). These data suggest that deguelin impacts IE gene transcription at times consistent with the transition to E and L transcription and the drug treatment impact on E and L viral gene transcription is more pronounced.

### 3.4. Deguelin Inhibits Viral Early Protein Expression

**Figure 6:**
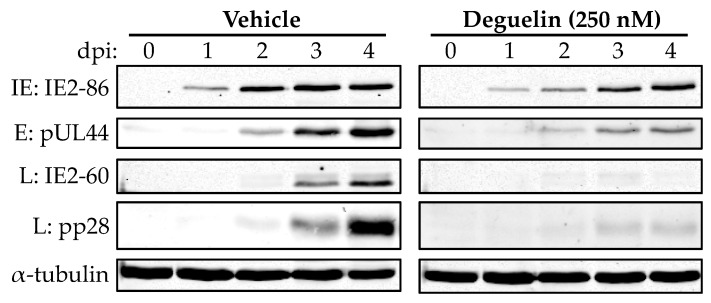
Deguelin treatment results in a reduction of E protein translation in HCMV infected fibroblasts. NuFF-1 cells were treated with vehicle (DMSO) or deguelin (250 nM) for 1 h, after which cultures were infected with UL99*eGFP* (moi = 1.0). Total cell lysates were collected over 4 d and viral protein expression was assessed by immunoblot for the following proteins: IE2-86 (IE), pUL44 (E), pp28 (L), and IE2-60 (L). Tubulin is shown as a loading control. Data is from a representative experiment (*n* = 3).

To further dissect the stage of lytic replication impacted by deguelin treatment, we next asked if viral protein expression was impacted. We treated NuFF-1 cells with either vehicle (DMSO) or deguelin (250 nM) for 1 h and then infected these cells with UL99eGFP (moi = 1.0). We collected total protein lysates over a time course of 4 d and assessed a representative viral protein from each of the kinetic classes of viral proteins. Consistent with our RNA data (Figure 5), we found that E (pUL44) and L (pp28) protein expression was significantly decreased in the deguelin-treated cells compared to those treated with vehicle. Additionally, we found that IE2-86, an IE protein, exhibited slightly delayed expression in the deguelin-treated cells that eventually accumulated to the levels seen in the vehicle control treated infections. However, IE2-60, an IE2 protein isoform that accumulates at late times of infection, displayed a significant reduction in protein expression in deguelin-treated cells compared to those that received vehicle treatment (Figure 6). Taken together, these data suggest that deguelin impacts HCMV lytic replication at times consistent with E gene/protein production.

## 4. Discussion

In the current study, we have shown that the naturally-occurring rotenoid deguelin significantly inhibits HCMV lytic replication. Specifically, our results indicate that sub-micromolar levels of deguelin impacts HCMV lytic replication as early as viral E transcript and protein expression. In turn, viral DNA, viral L gene and protein expression, and the production of infectious virus are all significantly decreased compared to infected fibroblasts that were treated only with vehicle. Importantly, HCMV strains that are resistant to ganciclovir treatment remain sensitive to deguelin treatment. Collectively, our data reveal a potentially new therapy to inhibit HCMV lytic replication.

Deguelin has been studied for just over 20 years, and much of the work has focused on its potential use as an anti-cancer agent. More specifically, investigators have found deguelin displays anti-cancer activities both in vitro and in vivo for a variety of cancers across tissue types, including non-small cell lung cancer [22,23,24,25], triple negative breast cancer [26,27], prostate cancer [28], gastric cancer [29,30,31], hepatocellular carcinoma [32,33], esophageal squamous cell carcinoma [34,35], acute myeloid leukemia [36,37,38,39], pancreatic cancer [40,41,42], head and neck squamous cell carcinoma [43,44,45], lung squamous cell carcinoma [46], and androgen receptor-positive breast cancer [47]. Deguelin induces apoptosis in non-small cell lung cancer cells, for example, by repressing Noxa through B lymphoma Mo-MLV insertion region 1 homolog (BMI1) regulation [25]. This compound also down-regulates hexokinase II-mediated glycolysis in these cancer cells [23], suggesting that deguelin functions are multifaceted. Deguelin also targets multiple signaling pathways, including Wingless-related integration site (Wnt)/β-catenin, nuclear factor kappa-light-chain-enhancer of activated B cells (NFκB), and PI3K/Akt, all of which are pro-survival pathways. Coupled with its ability to induce pro-apoptotic factors, deguelin clearly inhibits both cell survival and cell proliferation. In the case of lung squamous cell carcinoma cells, deguelin induces apoptosis via regulating galectin-1 expression [46]. In other settings, such as hepatocellular carcinoma cells, deguelin attenuates hepatocyte growth factor (HGF)-mediated HGF receptor (a.k.a. c-Met) activation, leading to vascular endothelial growth factor (VEGF) suppression, thereby suppressing angiogenesis and reducing pro-angiogenic factors in these cells [33]. In line with this observation, several groups have reported that deguelin inhibits hypoxia inducible factor-1α (HIF-1α) [48], a cellular protein overexpressed in many cancers. HIF-1α promotes tumor growth and metastases by promoting angiogenesis and regulating host cell metabolism. Like its function in regulating apoptosis, deguelin uses multiple mechanisms to inhibit angiogenesis. The ability of this compound to target multiple pathways involved in the progression of cancer has made deguelin an attractive, novel anti-cancer treatment.

As discussed above, deguelin inhibits a variety of signaling pathways that ultimately lead to the inhibition of cell survival and proliferation. Interestingly, many viruses, including HCMV, require many of these same factors that deguelin inhibits. For example, HCMV activates both the PI3K and mammalian target of rapamycin (mTOR) complex 1 (mTORC1) pathways [49], both of which are inhibited by deguelin [45,47,50]. In line with these data, HCMV stimulates survivin by inducing IL-6 [51], while deguelin inhibits survivin [12], a downstream target of the mTOR signaling [52]. During lytic replication, HCMV activates NFκB signaling, via upregulation of IκB kinases (IKKs), and in fact activation of this pathway is critical for activating the HCMV major immediate early promoter (MIEP) [53]. Deguelin, however, inhibits NFκB signaling and its downstream regulated genes by suppressing IKKs [54]. Similarly, as mentioned above, deguelin inhibits VEGF [33,48], HIF-1α [48], and Wnt/β-catenin [55], all of which HCMV activates during lytic replication [56,57,58,59,60]. Since deguelin inhibits many of the same pathways HCMV activates, it is attractive to speculate that the underlying mechanism of deguelin inhibition of HCMV lytic replication is multifaceted. Targeting cellular factors rather than those that are viral is a benefit, as most anti-viral compounds are directed against a specific viral protein. This is in contrast to anti-cancer therapies, which are directed against a host protein or pathway. Host-directed targeting as an antiviral therapeutic is attractive, as it limits viral resistance accumulation and has the capacity to target a wide range of viruses from distinct families. We propose that deguelin possesses antiviral characteristics and may serve as a suitable substrate for the development of novel antiviral compounds with pharmacological properties suitable for drug development.

## 5. Conclusions

In summary, the naturally occurring rotenoid, deguelin, significantly inhibits HCMV lytic replication. Importantly, we have shown that deguelin is effective at inhibiting ganciclovir-resistant HCMV, and thus may prove a suitable alternative therapeutic to treat multi-drug resistant HCMV.

## Figures and Tables

**Table 1:** Primers used in this study.

Primer Use	Sequence	Primer Name
galK insertion ^1^	CAACGTCCACCCACCCCCGGGACAAAAAAGCCCGCCGCCCCCTTGTCCTTTCCTGTTGACAATTAATCATCGGCA	UL99galK 5′
galK insertion ^1^	GTGTCCCATTCCCGACTCGCGAATCGTACGCGAGACCTGAAAGTTTATGAGTCAGCACTGTCCTGCTCCTT	UL99galK 3′
Reversion primer	CAACGTCCACCCACCCCCGGGACAAAAAAGCCCGCCGCCCCCTTGTCCTTTGTGAGCAAGGGCGAGGAGCTGTTCACCG	UL99eGFP 5′
Reversion primer	GTGTCCCATTCCCGACTCGCGAATCGTACGCGAGACCTGAAAGTTTATGAGTTACTTGTACAGCTCGTCCATGCCGAGAGT	UL99eGFP 3′
qPCR/RTqPCR	ATGGTTTTGCAGGCTTTGATG	UL122 FOR
qPCR/RTqPCR	ACCTGCCCTTCACGATTCC	UL122 REV
RTqPCR	GCCTTCCCTAAGACCACCAAT	UL123 FOR
RTqPCR	ATTTTCTGGGCATAAGCCATAATC	UL123 REV
RTqPCR	GGC GTG AAA AAC ATG CGT ATC AAC	UL44 FOR
RTqPCR	TAC AAC AGC GTG TCG TGC TCC G	UL44 REV
RTqPCR	CCCTCGGCTTCTCACAACAAT	UL54 FOR
RTqPCR	CGAGTTAGTCTTGGCCATGCAT	UL54 REV
RTqPCR	CACACAACACCGTCGTCCGATTAC	UL32 FOR
RTqPCR	GGTTTCTGGCTCGTGGATGTCG	UL32 REV
RTqPCR	GTGTCCCATTCCCGACTCG	UL99 FOR
RTqPCR	TTCACAACGTCCACCCACC	UL99 REV
RTqPCR	ACCCACTCCTCCACCTTTGAC	GAPDH ^2^ FOR
RTqPCR	CTGTTGCTGTAGCCAAATTCGT	GAPDH REV
qPCR	CCCCTTCCATCACATTGCA	MDM2 ^3^ FOR
qPCR	AGTTTGGCTTTCTCAGAGATTTCC	MDM2 REV

^1^ Underlined sequences correspond to the pGalK sequences. ^2^ GAPDH, glyceraldehyde 3-phosphate dehydrogenase. ^3^ MDM2, mouse double minute 2 homolog.

## Supplementary Materials

Supplementary File 1
